# Sub-population identification of multimorbidity in sub-Saharan African populations

**DOI:** 10.1038/s41598-025-96569-4

**Published:** 2025-04-22

**Authors:** Adebayo Oshingbesan, Michelle Kamp, Phelelani Thokozani Mpangase, Kayode Adetunji, Samuel Iddi, Daniel Maina Nderitu, Tanya Akumu, Okechinyere Achilonu, Isaac Kisiangani, Theophilous Mathema, Girmaw Tadesse, F. Xavier Gomez-Olive, Chodziwadziwa Whiteson Kabudula, Scott Hazelhurst, Gershim Asiki, Michele Ramsay, Skyler Speakman

**Affiliations:** 1 IBM Research Africa, Nairobi, Kenya; 2 Sydney Brenner Institute for Molecular Bioscience, Faculty of Health Sciences, University of the Witwatersrand, Johannesburg, South Africa; 3 African Population Health Research Center, Nairobi, Kenya; 4 MRC/Wits Rural Public Health and Transitions Research Unit (Agincourt), School of Public Health, Faculty of Health Sciences, University of Witwatersrand, Johannesburg, South Africa; 5 School of Electrical and Information Engineering, University of Witwatersrand, Johannesburg, South Africa; 6 Division of Epidemiology and Biostatistics, School of Public Health, University of Witwatersrand, Johannesburg, South Africa; 7 Social Genetic and Developmental Psychiatry Centre, Institute of Psychiatry, Psychology and Neuroscience, King’s College, London, UK

**Keywords:** Multimorbidity, Africa, Exploratory analysis, Survey data, Subset scanning, Scientific data, Comorbidities, Risk factors

## Abstract

This work provides three contributions that straddle the medical literature on multimorbidity and the data science community with an interest on exploratory analysis of health-related research data. First, we propose a definition for multimorbidity as the co-occurrence of (at least) two disease diagnoses from a pre-determined list. This interpretation adds to a growing body of working definitions emerging from the literature. Second, we apply this novel outcome of-interest to two sub-Saharan populations located in Nairobi, Kenya and Agincourt, South Africa. The source data for this analysis was collected as part of the Africa Wits-INDEPTH Partnership for Genomic Studies project. Third, we stratify this outcome-of-interest across all possible sub-populations and identify sub-populations with anomalously high (or low) rates of multimorbidity. Critically, the automatic stratification approach emphasizes efficient, disciplined exploratory-based analysis as a complementary alternative to more commonly-used confirmation analysis methods. Our results show that high-risk sub-populations identified in one part of the continent transfer to the other location (and vice-versa) with the equivalent sub-population at the other location also experiencing higher rates of multimorbidity. Second, we discover a real-world scenario where a more-at risk sub-population existed beyond the simpler sub-populations traditionally stratified by age and sex. This is in contrast to existing literature which commonly stratifies disease diagnoses by sex when reporting results. Patterns in diseases, and healthcare more generally, are likely more nuanced than manual approaches may be able to describe. This work helps introduce public health researchers to data science methods that scale to the size and complexity of modern day datasets.

## Introduction

Multimorbidity (MM), defined as the simultaneous occurrence of multiple chronic conditions within an individual, poses a significant global health challenge, contributing to increased mortality^1^, reduced quality of life^2^, and higher healthcare demand^3^. The global rise in the prevalence of MM is mirrored in Africa, fueled by aging populations and an increase in lifestyle risk factors such as obesity and physical inactivity^4,5^. The impact of MM in the region is further exacerbated by infectious diseases, poverty, and scarce healthcare resources^6^.

Research on MM has predominantly focused on populations of European ancestry or those in high-income countries, resulting in limited generalizability of findings to diverse populations and African populations being largely underrepresented. Studies that do include African-ancestry populations focus on African Americans, who are known to be poorly representative of the diverse populations of continental Africa^6–8^. Moreover, there are distinct MM patterns in continental and diaspora African-ancestry populations that call for representative and context-specific research to understand the true burden of MM among Africans residing in Africa^9^.

The multifaceted nature of healthcare within Africa, and particularly in the context of MM, necessitates a complex and nuanced approach to data analysis. While traditional statistical methods such as logistic, Cox, and Poisson regression have been valuable in identifying risk factors for MM^10,11^, their linear approach limits their ability to uncover complex interactions within specific sub-populations^7–9^. This limitation represents a significant gap in our understanding of MM. While interactions can be added manually, determining which interactions to include is challenging, and manually incorporating all potential interactions does not scale well^12^. This complexity leads to practical issues, such as high false discovery rates and “p-hacking”^13,14^.

**Fig. 1:**
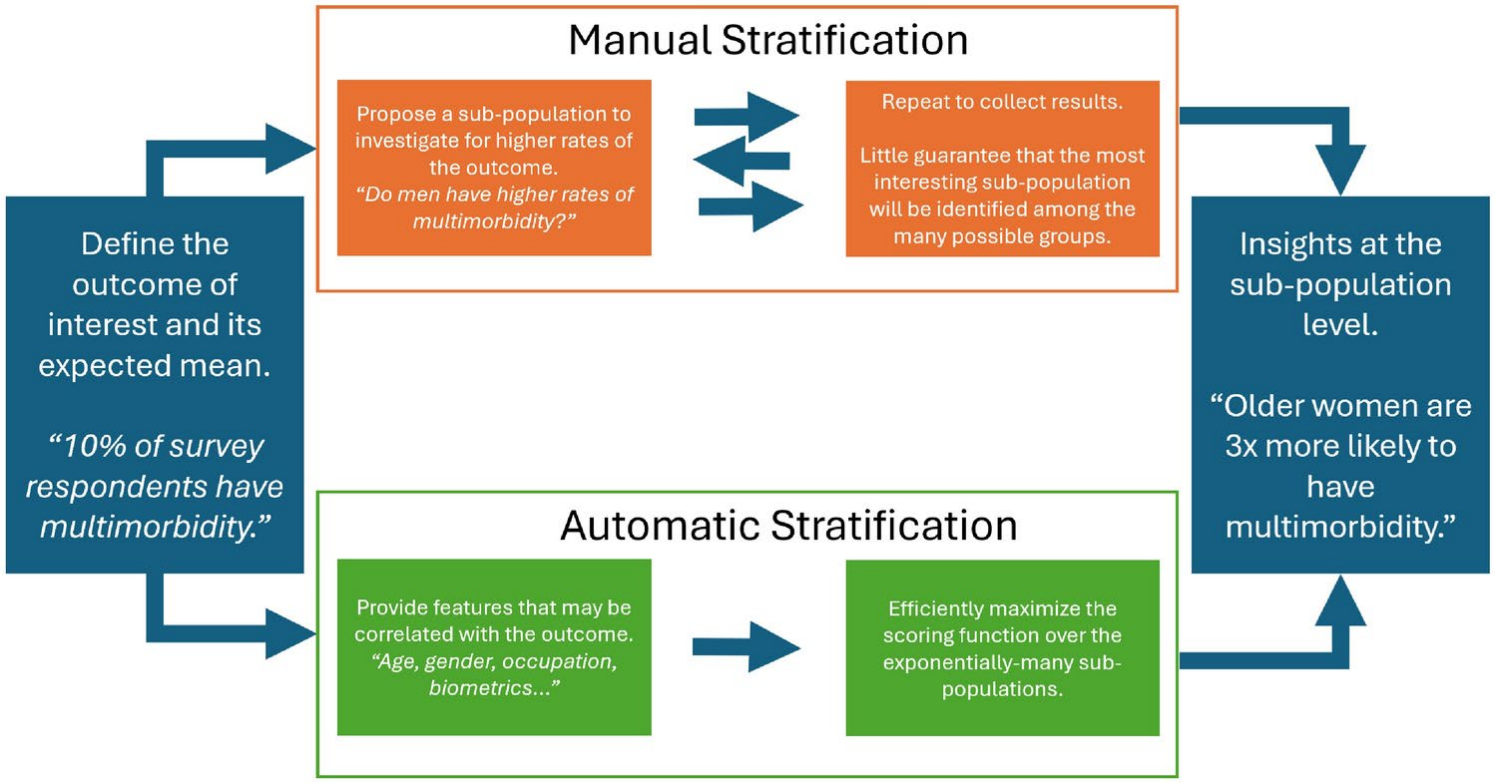
Flowchart illustrating the analysis pathways of manual and automatic stratification methods. Both approaches have the same initial data and end-goals. However, automatic stratification uses additional data science techniques to bring discipline and scalability to the otherwise slower manual approach. Also, manual stratification is more confirmatory in nature in that it measures evidence for a proposed hypothesis. Automatic stratification is more exploratory-driven in nature in that it finds hypothesis backed with the most evidence from the data. All analysis methods must recognize this balance^15^.

In this paper we demonstrate automatically stratifying the MM outcome across exponentially-many subsets of the dataset and reporting the extreme scenarios where MM is much higher (or lower) than expected. This is to assist domain experts in identifying interactions between (possibly many) features and the MM outcome in a disciplined manner. This approach differs from the more-common manual stratification by targeting discovery-driven analysis rather than a more hypothesis-based analysis as shown in Fig. 1. However, both manual and automatic stratification share the same goal of gaining insights of the outcome at the sub-population level. By leveraging the mathematical properties of divergence measures (likelihood ratios) and employing computational algorithms, we aim for a nuanced understanding of MM across sub-populations in two African cohorts.

## Related work

Age and sex are seminal variables in multimorbidity research, with a plethora of evidence from traditional quantitative analysis underpinning their significance^16^. The systematic review on aging and multimorbidity by Marengoni et al.^17^ adds another layer to the understanding. With a range of prevalence between 55% to 98% among older individuals, their exhaustive synthesis unearthed associations with etiological factors such as low socioeconomic status. This contribution increases the need for specialized care models, focusing on the multifactorial nature of multimorbidity in aging. Furthermore, a longitudinal study by Abad-Diez et al.^18^ and the comparative analysis of multimorbidity from survey data across 28 countries from Afshar et al.^19^ provided empirical insights into the role of age and sex differences in multimorbidity patterns, underscoring an elevated prevalence among older women.

A cross-sectional study conducted in the AWI-Gen cohort from the urban slums of Nairobi^20^, defined 14 chronic conditions and delineated the prevalence and determinants of lifetime multimorbidity (having two or more chronic conditions) as 28.7%, uncovering intricate associations with various socio-demographic and environmental factors. Their work provides essential evidence, elucidating the need for focused interventions within urban settings and highlighting the urban-rural divide’s nuanced implications. Contrasting this^21^, targeted their investigation toward preventive measures against multimorbidity within Low and Middle Income Countries (LMICs), emphasizing lifestyle interventions. Their methodological approach in identifying clusters of conditions like hypertension, diabetes, and cardiovascular disease lays the groundwork for early prevention strategies, fostering a paradigm shift towards pro-active public health initiatives.

Furthermore^22^, offers insights into the prevalence and correlates of multimorbidity (defined as two or more out of seven chronic conditions) in middle-aged men and women from the AWI-Gen study across four sub-Saharan African countries, revealing geographical variations in multimorbidity with South African men and women having the highest rates of multimorbidity (51.7% and 64.9%, respectively), followed by East Africa (31.3% and 48.4%) and then West Africa (20.2% and 24.1%). While, age and body mass index (BMI) were identified as significant risk factors for both sexes, lifestyle factors such as alcohol consumption had different impacts on men and women.

Importantly, each study design used a different set of chronic conditions (sometimes even from data from the same cohort) to define multimorbidity, limiting direct comparison between studies. The differences in the outcomes between studies illustrate the need for a careful description of the data, the definition of multimorbidity and the objectives of each of the studies. None of those mentioned above used automated and scalable techniques to identify sub-populations with a higher proportion of individuals affected by multimorbidity. Recent works in the domain of sub-population analysis have shown the importance of understanding health data at the sub-population level, discovering relationships that purely domain-driven analysis may miss^23–26^.

## Method

The core methodology of this work provides an example of automatically stratifying an outcome of interest over exponentially-many sub-populations with a goal of discovering where (in the dataset) the sub-populations with extremely high (or low) rates of the outcome are. The goals of this approach are very similar to to the goals of *manual* stratification: to gain insights about the outcome at a sub-population level. Both approaches to stratification (manual and automatic) are complementary alternatives to classical regression methods which look for patterns spanning the entire dataset.

### Study design and setting

The analysis in this work is from the first wave of data collection from the Africa Wits-INDEPTH Partnership for Genomic Studies project, referred to as AWI-Gen^27,28^. We specifically look at two groups of respondents from Agincourt, South Africa and Nairobi, Kenya. Multi-morbidity is defined as having two or more of the following conditions: hypertension (HT), diabetes mellitus (DM), chronic kidney disease (CKD), and cardiovascular disease (CVD). The AWI-Gen dataset provides a unique opportunity to explore differences in multi-morbidity patterns across geographic locations and demographic groups. Agincourt and Nairobi were selected as the study sites due to their diverse populations and varying health contexts.

### Data collection, measurements, and definition of variables

The dataset is carefully curated to include only individuals aged 40 to 60, ensuring that the selected variables are relevant and properly quality-controlled. The primary outcome variable is the presence of multi-morbidity, defined as having at least two of the specified conditions (HT, DM, CKD, and CVD). These conditions are determined based on participants’ medical histories, considering ever-diagnosed status to account for the lack of control information.

Variables encompass both continuous and categorical factors. Continuous variables include BMI (kg/m2), age at data collection (years), carotid intima-media thickness (CIMT (mm)), lipid levels (TC, LDL-C, HDL-C, triglycerides (mmol/L)), waist-to-hip ratio, waist circumference (mm), visceral adipose tissue (VAT), and subcutaneous adipose tissue (SCAT). Categorical variables comprise sex, HIV status, education level, partnership status, socioeconomic status (SES) quintile, employment/occupation, moderate-vigorous intensity physical activity (MVPA), alcohol consumption, and smoking status.

In Agincourt, there were a total of 1465 participants between the ages of 40 and 60. However, only 1377 participants (94%) had all data for HT, DM, CKD, and CVD. On the other hand, for Nairobi, there were a total of 1942 participants with 1777 participants (92%) having all data for HT, DM, CKD, and CVD. Stratified by site, missing was $\le$1% for 16 out of the 20 variables in Agincourt and all 20 variables in Nairobi (see Supplementary Table S1 for exact missing percentages per variable).

### Definition of multimorbidity

Each of these outcome variables hypertension, diabetes mellitus, chronic kidney disease, and cardiovascular disease are carefully defined, and a consistent set of criteria is applied to ensure comparability and reliability. The conditions are assessed based on participants’ medical histories and self-reported information, acknowledging the limitations of relying solely on historical diagnoses without considering the current control status of the conditions. This approach is particularly relevant in the absence of accurate data regarding the control of these conditions.

The definitions of the individual outcome variables are as follows:

1. Hypertension (HT): Hypertension refers to elevated blood pressure levels consistently measured above the normal range. Hypertension was defined as systolic blood pressure $\ge$140 mm Hg and/or diastolic blood pressure $\ge$ 90 mmHg, in line with the seventh report of the Joint National Committee on Prevention, Detection, Evaluation, and Treatment of High Blood Pressure^29^, or if the participant was taking hypertension medication. It is a well-established condition for various health complications.
2. Diabetes Mellitus (DM): Diabetes mellitus is characterized by elevated blood glucose levels due to inadequate insulin production or impaired insulin utilization. It includes both type 1 and type 2 diabetes. Diabetes was defined using the WHO criteria, which are the presence of a previous diagnosis of diabetes by a healthcare professional or fasting blood glucose $\ge$ 7 mmol/L or random glucose $\ge$ 11.1 mmol/L^30^, or on diabetes medication at the time of recruitment.
3. Chronic Kidney Disease (CKD): Chronic kidney disease involves the progressive decline of kidney function over time, often leading to impaired filtration and regulation of bodily fluids. CKD is associated with a range of adverse health outcomes. CKD was defined as estimated glomerular filtration rate (eGFR) $\le$ 60 mL/min per 1.73 m2 (calculated using the Chronic Kidney Disease Epidemiology (CKD-EPI) (creatinine) equation 2009, without adjustment for African American ethnicity), presence of albuminuria (urine albumin creatinine ratio>3 mg/mmol) or both^31^. As the study was cross-sectional, low eGFR and albuminuria were not confirmed with follow-up testing.
4. Cardiovascular Disease (CVD): Cardiovascular disease encompasses various conditions affecting the heart and blood vessels, such as coronary artery disease, heart failure, and stroke. CVD was defined as present if the participant reported having had a heart attack or stroke or transient ischaemic attack. Participants previously diagnosed with congestive heart failure or angina were also classified as having CVD.

These conditions were selected based on their individual impact on health and the potential synergistic effects when they co-occur. The study acknowledges the complexity of multi-morbidity and the challenges in differentiating between individuals who have controlled their conditions and those who still experience active health concerns. Due to limitations in data availability and accuracy, this study focuses on capturing the presence of these chronic conditions without delving into the control status of each individual condition. UpSet plots are used for visualizing the data described, as they enable the analysis of complex relationships and intersections among the four conditions selected in our dataset^32,33^. These plots help delineate the prevalence and coexistence of conditions in the population studied. While techniques like UpSet plots and traditional quantitative analysis helps us understand the co-occurences of conditions with one another and with features, they do not enable us get a more nuanced sub-population view.

### Automatic stratification

Stratifying an outcome of interest across different sub-populations is one of the earliest forms of data analysis. Stratification aids investigators in understanding the distribution of the outcome among “old vs. young” or “male vs. female” participants, for example. Often, the investigators are looking for a subset of the population with much higher (or lower) rates of the outcome as compared to the average over the entire dataset.

Although intuitive, the *manual* process of identifying subsets with higher (or lower) outcomes leaves a lot to be desired. First, investigators will typically only explore sub-populations stratified across a single feature at a time (e.g. “age”). In large, complex healthcare datasets these presupposed strata are likely too simplistic to capture the true underlying subset. Second, manual stratification typically lacks discipline in comparing subsets to each other. Neither of these issues are a concern if the goal is to only explore a small number of pre-defined sub-populations in the data. However, in order to scale the stratification goals to modern-day datasets, we must use tools that address these limitations of the manual process. Automatic stratification is a data exploration technique that brings discipline and scalability to the goals of manual stratification.

The discipline of automatic stratification comes from its objective scoring function that quantifies the statement: “higher-than-expected outcomes of interest.” The scalability of automatic stratification comes from its ability to *maximize* this scoring function over exponentially-large search space of sub-populations. We now explore these two concepts in more detail.

#### Discipline from the scoring function 

Automatic stratification uses a likelihood ratio based on the Binomial Distribution to quantify the anomalousness of multimorbidity counts observed in a subset, *S*. Let *C*(*S*), be the observed number (count) of multimorbidity cases in a subset, *S*, and let *B*(*S*) be the expected (baseline) number of multimorbidity cases in that same subset. The expected number of multimorbidity cases in a subset can be directly calculated by scaling the *average* multimoribidity rate in the entire population by the size of the subset, |*S*|. The scoring function, *F*(*S*), measures the divergence between *C*(*S*) and *B*(*S*), providing a quantifiable measure of “higher-than-expected” rates of multimorbidity in a subset. Intuitively, *F*(*S*) should be large when *C*(*S*) $>>$ *B*(*S*). However, the size of the subset, |*S*|, also impacts the score. Equation 1 is derived from the likelihood ratio of two binomial distributions and simplified to rely only on the observed, *C*(*S*), counts of multimorbidity and the expected, *B*(*S*), counts of multimoribidity in a subset of the population, *S*, containing |*S*| records.

1 $$\begin{aligned} F(S)&= C(S) \cdot \log {\left( \frac{C(S)}{B(S)} \right) } + (|S| - C(S)) \cdot \log {\left( \frac{|S|-C(S)}{|S|-B(S)} \right) } \end{aligned}$$

The natural log is is written as log for readability. Additional background and motivation for this scoring function are provided in^24^.

#### Scalability from efficient maximization

Automatic stratification seeks to identify the highest scoring (most anomalous) sub-population among the exponentially-many to consider. Using an appropriate scoring function, the goal is to identify $S^*$ where

2 $$\begin{aligned} S^* = \arg \max _{S \in \mathcal {S}} F(S) \end{aligned}$$

and $\mathcal {S}$ is the search space of all possible subsets.

Automatic stratification is a specialized version of the more general Multi-dimensional Subset Scan (MDScan)^34^. MDScan was initially developed in the spatial-temporal epidemiology context to identify geographic regions with higher-than-expected disease counts. In this work we do not use spatial or temporal features to identify higher disease counts, but rather focus on clinical, familial, and socio-economic features. Second, automatic stratification uses a more restrictive definition of expected counts, *B*(*S*), based on the mean of the outcome of interest in the dataset. Whereas MDScan allows the expected number of counts to be based on a recent time-window or a predictive function.

MDScan is an iterative ascent procedure (see Supplementary Algorithm S1 for pseudocode) where each step is efficient and exact due to the Additive Linear-time Subset Scanning property (ALTSS) ^35,36^ of commonly-used scoring functions. While a feature containing *k* unique values has exponentially many ($O(2^k)$) sub-populations, a scoring function that satisfies the ALTSS property guarantees that the most anomalous (highest scoring) sub-population will be one of only linearly-many (*O*(2*K*)) subgroups. This property of scoring functions makes this optimization problem tractable for datasets containing billions or trillions of possible subsets.

#### Complexity penalties

 Scaling the goals of stratification to cover exponentially-many sub-populations is a double-edged sword. On the one hand, automatic stratification can highlight subsets of the data that would not have been considered in a manual search. However, the subset, $S^*$, that maximizes the divergence between *C*(*S*) and *B*(*S*) (see Equations 1 and 2) may be obtuse due to too many features participating in the subset’s description^24^. Automatic stratification (and the underlying Multi-dimensional Subset Scanning algorithm) adds a complexity penalty to the scoring function that can give preference to “simpler” subsets. More formally, the complexity of a subset is measured by the number of literals used to describe the subset, $S_{NL}$. As examples, the sub-population (Sex = Female) is described using 1 literal ($S_{NL} = 1$) whereas the subset ( Sex = Female and Age $\ge$ 54 and BMI $\ge$ 23 and Waist Circumference $\ge$ 830 and Occupation = unemployed) uses 5 literals ($S_{NL} = 5$).

This measure of subset complexity is scaled linearly by a user-supplied complexity penalty, $\rho \ge 0$, and then subtracted from the original scoring function, *F*(*S*). This creates a *penalized* scoring function shown in Equation 3 that can give preference to simpler-to-describe subsets identified by automatic stratification.

3 $$\begin{aligned} F_{pen}(S) = F(S) - \rho S_{NL} \end{aligned}$$

The parameter $\rho$ can be thought of as a regularization term in the optimization procedure underlying automatic stratification. By changing the strength of $\rho$, investigators can explore sub-populations of varying description lengths as shown in our Results section. Lastly, $F_{pen}(S)$ can replace *F*(*S*) in Equation 2 while still allowing efficient maximization due to both scoring functions satisfying the Additive Linear Time Subset Scanning Property^36^.

#### Significance testing

 To assess the statistical significance of the identified sub-population, we employ randomization-based hypothesis testing ^37,38^. This choice is motivated by the exponential nature of the hypothesis testing involved in the discovery process. Traditional correction methods like the Benferroni correction ^39^ and the Benjamini-Hochberg correction ^40^ are overly conservative and may erroneously label many discovered subgroups as false positives. For randomization-based hypothesis testing, we perform $K = 100$ iterations. In each iteration *k*, we generate a synthetic version of the original dataset but with the outcome for each record replaced by a weighted coin toss (the weight of the coin toss comes from the population mean of the outcome). This synthetic (sometimes called replica) dataset represents the null hypothesis assumption that *every* subset in the data has outcomes drawn from the population mean. We then apply automatic stratification to each replica dataset, yielding divergence scores for all replicas ($F(S_k)$, $k = 1,2, \ldots , 100$), which are then compared to the true divergence score ($F(S^*)$). An empirical *p*-value (*p*) is computed as $p = (r(S^*)+1) / (K+1)$, where $r(S^*) = \sum _{k=1}^{K}{\zeta _k(S^*)}$, and $\zeta _k(S^*) = 1$ if $F(S_k) \ge F(S^*)$, otherwise $\zeta _k(S^*) = 0$.

## Results and discussion

### Understanding co-occurrences of health conditions

**Fig. 2:**
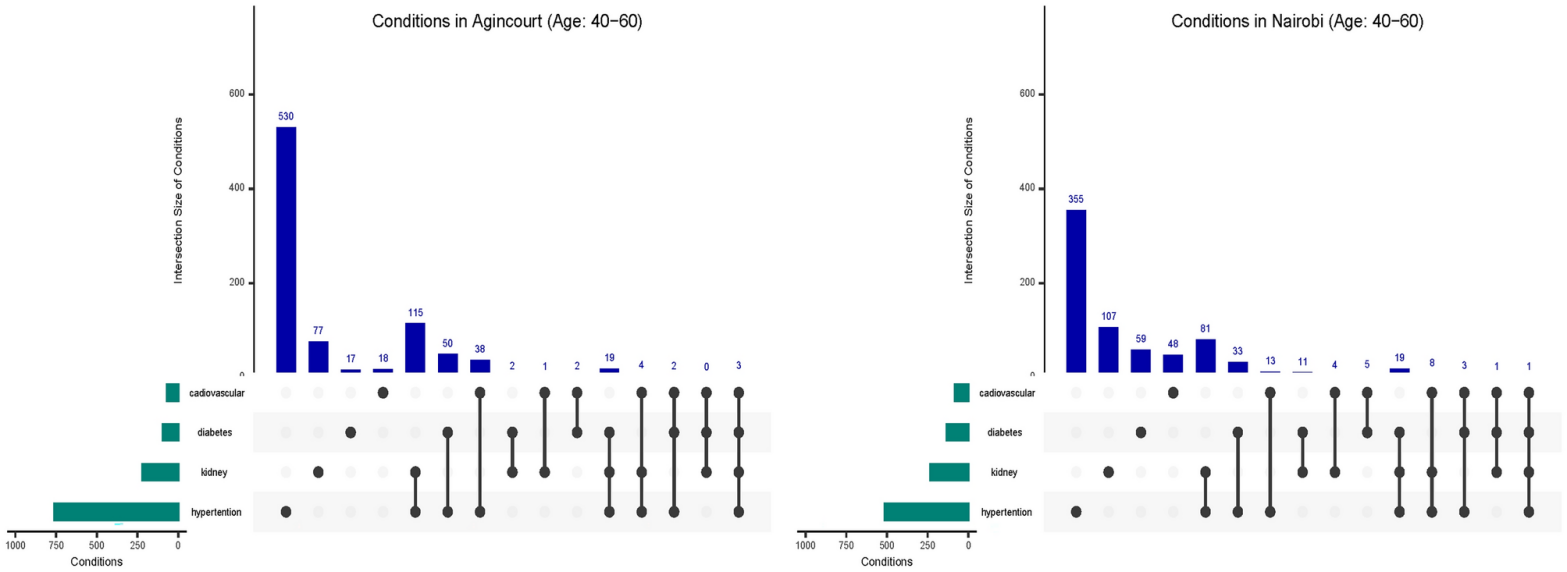
Upset Plots illustrating the co-occurrence of four health conditions from Agincourt and Nairobi.

Upset Plots in Fig. 2 show that Hypertension is the predominant health condition in both Agincourt and Nairobi locations with Agincourt notably higher than Nairobi. Our working definition of multimorbidity results in a MM prevalence of 16.4% in Agincourt and 9.7% in Nairobi. Furthermore, the co-occurrence of HT with CKD is a higher burden in Agincourt than it is in Nairobi. In fact, the co-occurrence of HT and CKD in Agincourt is more frequent than the incidence of CKD alone. Despite the disparity in HT rates, the overall pattern of disease co-occurrence in Agincourt mostly aligns with that observed in Nairobi, suggesting potential commonalities in disease interplay between the two geographies. The similarity in co-occurrence patterns might be indicative of shared epidemiological or socio-environmental factors that govern disease dynamics in these populations.

### Sub-population discovery using automatic stratification

Tables 1 and 2 present the stratified sub-populations in Agincourt and Nairobi, identified through automated stratification. Both high and low risk subsets of varying complexity are provided. Subset complexity is measured by the number of literals used to describe the subset (NL). We report subpopulations that contained between 2 and 4 NL in their descriptions. The lower bound is because subsets with 1 literal may be easily identified through an exhaustive search and lack interactions across multiple features. The upper bound is due to subpopulation descriptions becoming too opaque for domain experts to interpret. In addition to the description and number of literals in the description, the tables also provide the following descriptive statistics of each discovered sub-population.

- Odds Ratio- (OR). The numerator is the odds of having mulimorbidity for participants in the subset and the denominator is the odds of having multimorbidity for participants outside the subset.
- Relative Size of the Subset- P(S). This value is written as the probability of a participant being in the subset. Multiplying this value by the total population size would result in the absolute size of the subset, |*S*|.
- Multimorbidity Rate Within the Subset- P(MM$\vert$**S)**. This value is written as a the probability of a participant in the subset having multimormobdity. These values should drastically diverge away from the baseline rates of MM in each location.
- Proportion of Multimorbidity Contained in the Subset- P(S$\vert$MM). This value is written as the probability that a participant with multimorbidity is in the subset. These values should diverge away from the relative size of the subset, P(S).

We now take a closer look of these values for rows 1 and 3 from Agincourt in Table 1. Row 1 has a relatively simple subset description with 2 literals describing participants over the age of 53 and a waist circumference exceeding 950mm. This group is much easier to describe than row 3 which has a subset description requiring 4 literals. Returning to row 1, participants in this group are 3.57 times more likely to have multimorbidity than others in Agincourt. Furthermore, although this sub-population contains 16.4% of Agincourt participants, it contains nearly 35% of all multimorbidity cases in Agincourt.

By decreasing the complexity penalty used in the optimization process, automatic stratification can return more complex subsets with longer description lengths. Like most regularization techniques, there is a tension between interpretability of the subset(i.e., shorter description lengths) and the amount of divergence between the observed and expected number of multimoribidity cases within a subset. The appropriate number of literals used to describe a subset can be domain or even investigator-specific. Therefore, we encourage analysts to consider a broad range of complexity penalties and observe how the anomalous subsets change with their description lengths.

For this analysis, we turn our focus to the Nairobi location and consider rows 1 and 2 from Table 2. The simpler subset with two literals in its description are women over the age of 54. The more complex subset (3 literals) changes to measurements for CIMT, triglycerides, and waist-hip ratio. Looking only at the subset descriptions, it is difficult to tell if these two subsets are describing overlapping or exclusive parts of the original overall population. Is this subset also describing older females but in a different form?

**Fig. 3:**
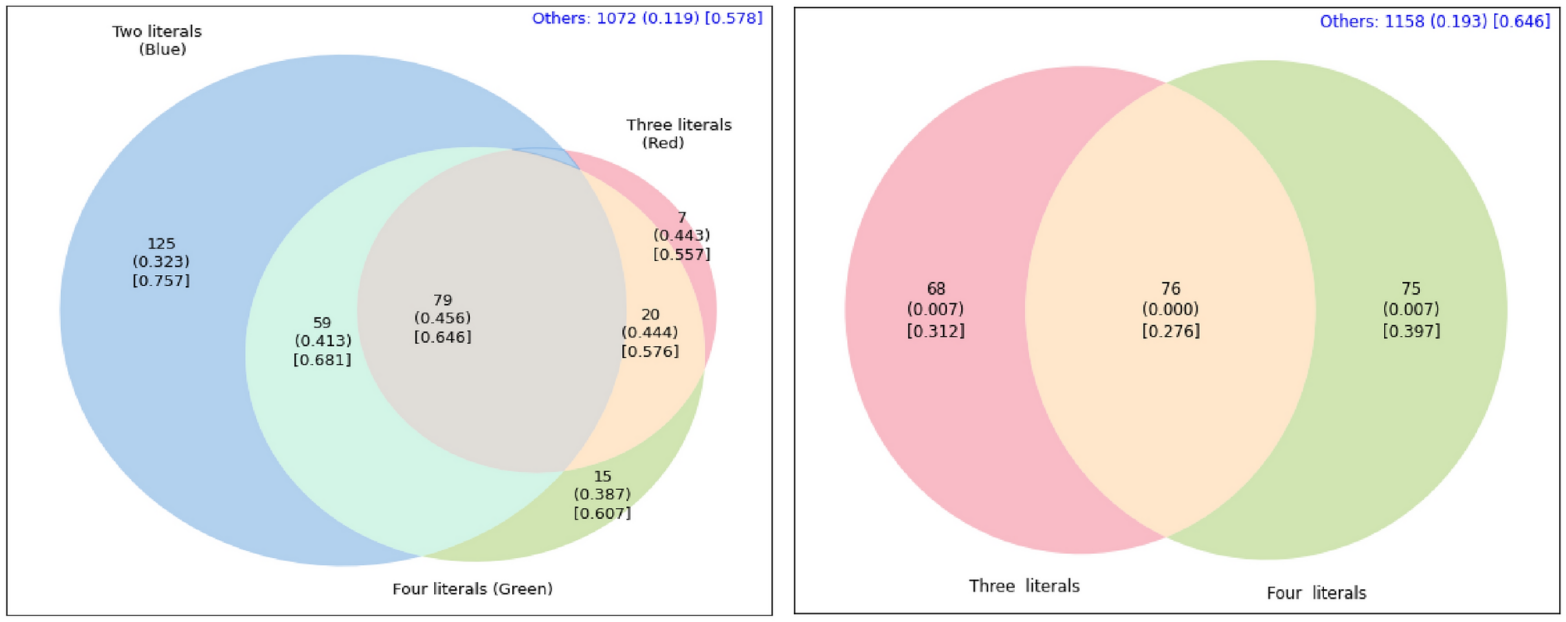
Venn Diagram for the sub-populations discovered by automatic stratification in Agincourt as described in Table 1. The left image is for sub-populations with High Risk Status and the right is for sub-populations with Low Risk Status. Each value in the Venn diagram represents the count of individuals in that section, the percentage of multimorbidity within that count in parenthesis, and the percentage of women in that subgroup is in square brackets. The blue text at the upper right corner describes the number of people that are not in any of the sub-populations participating in the Venn diagram and the percentage of multimorbidity within those.

**Fig. 4:**
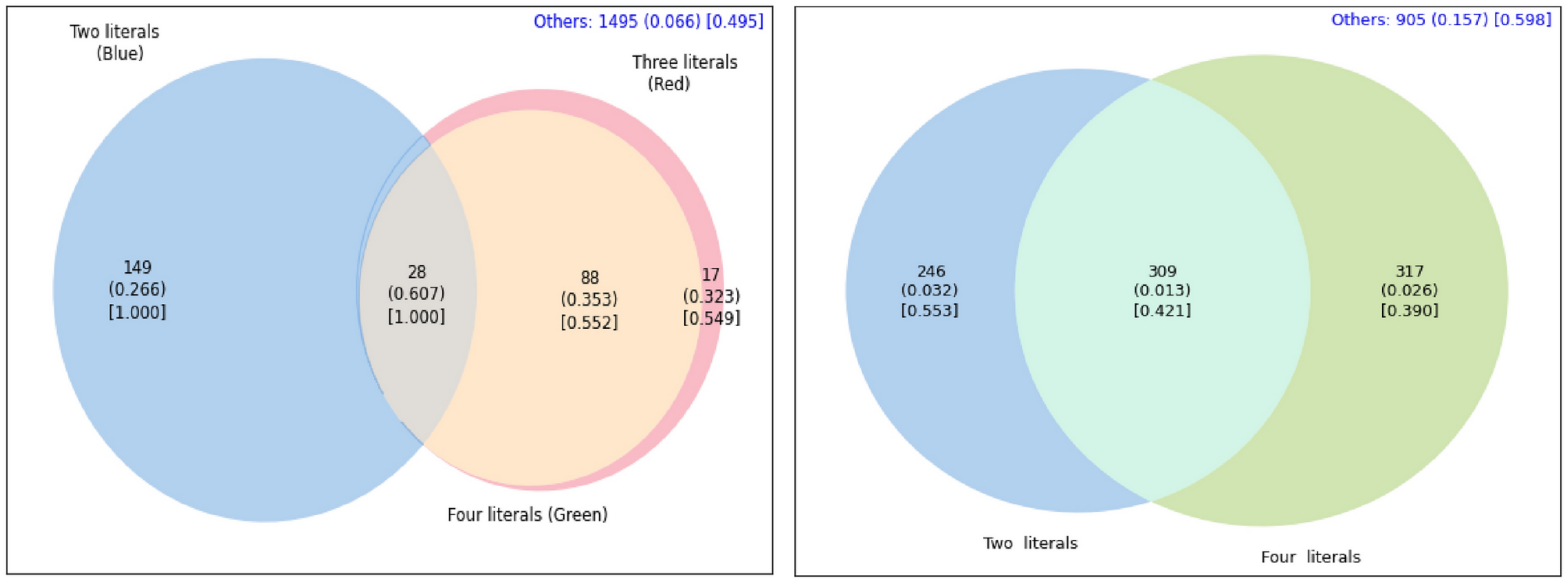
Venn Diagram for the sub-populations discovered by automatic stratification in Nairobi as described in Table 2. The left image is for sub-populations with High Risk Status and the right is for sub-populations with Low Risk Status. Each value in the Venn diagram represents the count of individuals in that section, the percentage of multimorbidity within that count in parenthesis, and the percentage of women in that subgroup is in square brackets. The blue text at the upper right corner describes the number of people that are not in any of the sub-populations participating in the Venn diagram and the percentage of multimorbidity within those.

Venn diagrams can aid in this exploration of anomalous subsets of varying description lengths. Figures 3 and 4 show the overlap (or lack thereof) of the sub-populations described in the Tables for both Agincourt and Nairobi locations, respectively.

For example, in the left side of Fig. 4 we observe that the two subsets described in rows 1 and 2 of Table 2 are capturing different populations with minimal overlap. These two subsets only share 28 participants in common while having 149 and 88+17=105 unique to each subset. This type of analysis is *not possible* with manual stratification approaches. Manual stratification would likely limit the search process to much simpler subsets such as sex = female (by itself) and age $\ge$ 53 (by itself). Manual stratification would miss the subset(s) with higher rates of multimorbidity because it lacks the scale and discipline provided by automatic stratification to efficiently explore exponentially-many sub-populations.

This subset discovery analysis was repeated under different conditions. In the first variation, we searched over a reduced search space by removing features that are more difficult to acquire outside of health survey settings. The resulting subsets in Supplementary Tables S2 and S3 may be more representative of what is available from typical health clinc data. Second, we stratified for anomalous subpopulations contained entirely within male and female populations. This can be interpreted as *forcing* the sex feature to be included in the anomalous subset returned by automatic stratification. Results for both locations and sex status can be found in Supplementary Tables S4-S7.

**Table 1:** Subpopulations of participants in Agincourt (n=1377; 16.4% multimorbidity) with high and low risk for multimorbidity as identified by automatic stratification.

Risk status	NL	Subpopulation description	OR	P(S)	P (MM|S)	P (S|MM)
High risk	2	Age $\ge$ 53 years & waist circumference $\ge$ 950 mm	3.57	0.164	0.345	0.345
	3	Age $\ge$ 53 years & CIMT mean max $\ge$ 0.72 mm & waist hip ratio $\ge$ 0.92	4.86	0.081	0.441	0.217
	4	Age $\ge$ 53 years & BMI $\ge$ 21.44 $kg/m^2$ & CIMT mean max $\ge$ 0.62 mm & waist hip ratio $\ge$ 0.92	4.25	0.118	0.395	0.283
Low risk	3	Visceral fat $\ge$ 4.33 mm & waist circumference $\le$ 780 mm & waist hip ratio $\le$ 0.92	0.04	0.084	0.009	0.004
	4	Age $\le$ 57.0 years & CIMT mean max $\le$ 0.66 mm & triglycerides $\le$ 1.14 mmol/L & waist circumference $\le$ 780 mm	0.04	0.092	0.008	0.004

NL refers to the number of literals present in the description, OR refers to the odds ratio of the outcome of interest for the described subpopulation. P(S) refers to the size of the subpopulation as a proportion of the overall size of the dataset. P(MM$\vert$S) refers to the proportion of the described subpopulation that have multimorbidity. P(S$\vert$MM) refers to the proportion of multi-morbid people that the described subpopulation covers. All found subsets were statistically significant at $p-value = 0.01$

**Table 2:** Subpopulations of participants in Nairobi (n=1777; 9.7% multimorbidity) with high and low risk for multimorbidity as identified by automatic stratification.

Risk status	NL	Subpopulation description	OR	P(S)	P (MM|S)	P (S|MM)
High risk	2	Age $\ge$ 54 years & sex is Female	4.91	0.084	0.295	0.256
	3	CIMT mean max $\ge$ 0.64 mm & triglycerides $\ge$ 1.05 mmol/L & waist hip ratio $\ge$ 0.94	5.82	0.073	0.331	0.250
	4	Age $\ge$ 43 years & CIMT mean max $\ge$ 0.64 mm & triglycerides $\ge$ 1.05 mmol/L & waist hip ratio $\ge$ 0.94	6.65	0.062	0.364	0.233
Low risk	2	Age $\le$ 46 years & waist hip ratio $\le$ 0.9	0.26	0.263	0.034	0.093
	4	Age $\le$ 54 years & BMI $\le$ 25.8 $kg/m^2$ & hdl $\le$ 1.53 mmol/L & waist hip ratio $\le$ 0.94	0.18	0.338	0.027	0.093

NL refers to the number of literals present in the description, OR refers to the odds ratio of the outcome of interest for the described subpopulation. P(S) refers to the size of the subpopulation as a proportion of the overall size of the dataset. P(MM$\vert$S) refers to the proportion of the described subpopulation that have multimorbidity. P(S$\vert$MM) refers to the proportion of multi-morbid people that the described subpopulation covers. All found subsets were statistically significant at $p-value = 0.01$

### Sub-population robustness: cross-location transferability

Do high-risk subsets discovered in Nairobi also describe a high-risk sub-population in Agincourt or vice versa? Are these subsets robust across locations? To answer these questions we employed cross-location transferability analysis. This method involves taking a sub-population delineated at a source location (e.g., Nairobi) and identifying the corresponding cohort at a target location (e.g., Agincourt) that meets the same description. The last step is to determine if the subset in the target location also shows evidence of significantly divergent odds ratios. This is done by calculating 95% confidence intervals for the odds ratio and determining if these intervals contain 1.0. Additional metrics, including sub-population size and the prevalence of multimorbidity, were also recorded. The results, displayed in Tables 3 and 4, indicate a successful cross-location validation for nine out of ten sub-populations identified by automatic stratification within high- and low-risk categories between both Agincourt and Nairobi sites. Notably, odds ratios for high-risk sub-populations reached up to 3.2, whereas low-risk counterparts were identified with odds ratios as low as 0.3. This performance contrasts sharply with sub-populations demarcated solely by sex, where odds ratios peaked at 1.6 and dipped to 0.6 across both sites. The lone outlier, a sub-population with high visceral fat yet classified as low risk in Agincourt-highlights unique regional data patterns and underscores the nuanced understanding of risk facilitated by stratification. This exception may signal specific regional health determinants or data-collection idiosyncrasies exclusive to the Agincourt cohort.

**Table 3:** Robustness of the subpopulations descriptions with high and low risk for multimorbidity in Agincourt as identified by automatic stratification when transferred to Nairobi.

Risk status	NL	Subpopulation description	OR	95 CI (low)	95 CI (high)	P (S)	P (MM|S)	P (S|MM)
High risk	2	Age $\ge$ 53 years & waist circumference $\ge$ 950 mm	4.19	2.71	6.48	0.067	0.277	0.192
	3	Age $\ge$ 53 years & CIMT mean max $\ge$ 0.72 mm & waist hip ratio $\ge$ 0.92	5.15	3.17	8.38	0.047	0.325	0.157
	4	Age $\ge$ 53 years & BMI $\ge$ 21.44 $kg/m^2$ & CIMT mean max $\ge$ 0.62 mm & waist hip ratio $\ge$ 0.92	4.87	3.11	7.62	0.059	0.308	0.186
Low risk	$3^*$	Visceral fat $\ge$ 4.33 mm & waist circumference $\le$ 780 mm & waist hip ratio $\le$ 0.92	0.77	0.44	1.35	0.101	0.078	0.081
	4	Age $\le$ 57 years & CIMT mean max $\le$ 0.66 mm & triglycerides $\le$ 1.14 mmol/L & waist circumference $\le$ 780 mm	0.49	0.26	0.9	0.118	0.053	0.064

NL refers to the number of literals present in the description, OR refers to the odds ratio of the described subpopulation. 95CI(low) and 95CI(high) provides the lower bound and upper bound of the OR respectively at a 95% confidence level. P(S) refers to the size of the subpopulation as a proportion of the overall size of the dataset. P(MM$\vert$S) refers to the proportion of the described subpopulation that have multimorbidity. P(S$\vert$MM) refers to the proportion of multimorbid people that the described subpopulation covers. $\{NL\}^*$ means subset transferability/robustness failed at 95 CI

**Table 4:** Robustness of the subpopulations descriptions with high and low risk for multimorbidity in Nairobi as identified by automatic stratification when transferred to Agincourt.

Risk status	NL	Subopulation description	OR	95 CI (low)	95 CI (high)	P(S)	P (S|MM)	P (MM|S)
High risk	2	Age $\ge$ 54 years & sex is Female	2.13	1.54	2.95	0.187	0.261	0.296
	3	CIMT mean max $\ge$ 0.64 mm & triglycerides $\ge$ 1.05 mmol/L & waist hip ratio $\ge$ 0.94	3.23	2.04	5.1	0.064	0.364	0.142
	4	Age $\ge$ 43 years & CIMT mean max $\ge$ 0.64 mm & triglycerides $\ge$ 1.05 mmol/L & waist hip ratio $\ge$ 0.94	3.79	2.37	6.09	0.058	0.4	0.142
Low risk	2	Age $\le$ 46 years & waist hip ratio $\le$ 0.9	0.3	0.17	0.52	0.16	0.063	0.062
	4	Age $\le$ 54 years & BMI $\le$ 25.8 $kg/m^2$ & hdl $\le$ 1.53 mmol/L & waist hip ratio $\le$ 0.94	0.46	0.3	0.7	0.211	0.093	0.119

NL refers to the number of literals present in the description, OR refers to the odds ratio of the described subpopulation. 95CI(low) and 95CI(high) provides the lower bound and upper bound of the OR respectively at a 95% confidence interval as a measure of the robustness of the transferability of the subpopulation. P(S) refers to the size of the subpopulation as a proportion of the overall size of the dataset. P(MM$\vert$S) refers to the proportion of the described subpopulation that have multimorbidity. P(S$\vert$MM) refers to the proportion of multi-morbid people in the entire population that the described subpopulation covers

## Conclusion

This study described and leveraged automatic stratification to discover high and low-risk sub-populations for a novel multimorbidity outcome in Agincourt, South Africa and Nairobi, Kenya. Using a more data-driven methodology than manual stratification, automatic stratification discovered nuanced sub-populations that may not have been evident through customary domain knowledge driven analysis such as sex-based analysis alone. Cross-site transferability analyses confirmed the robustness of the discovered subsets across different locations within Africa, validating the tool’s efficacy in global health contexts, and avoiding some of the generalization pitfalls that other data-driven discovery processes tend to suffer from.

However, the study’s definition of multimorbidity, while pragmatic, is constrained by data availability and the scope of conditions included. The choice to analyze multimorbidity based on the presence of two or more conditions rather than on a pairwise basis may have influenced the study’s outcomes and interpretability. Future work should involve a closer collaboration with domain experts to refine the definition of multimorbidity and expand its clinical applicability. By dissecting the condition-pairs prevalent in the target populations, future research could pave the way for advancements in the management of multimorbidity in African populations.

## Supplementary Information

Supplementary Information.

## Data Availability

The AWI-Gen data collection was funded by the National Human Genome Research Institute (NHGRI), the National Institute of Environmental Health Sciences (NIEHS), the Office of AIDS research (OAR) and the National Institute of Diabetes and Digestive and Kidney Diseases (NIDDK), of the National Institutes of Health (NIH) under award number U54HG006938, as part of the H3Africa Consortium, and by the Department of Science and Innovation, South Africa, award number DST/CON 0056/2014. The AWI-Gen data used in this manuscript is stored at the European Genome-Phenome Archive (EGA) https://ega-archive.org/datasets/EGAD00001006425.
