# Supplementary material for: Sub-population identification of multimorbidity in sub-Saharan African populations
Source: Sci Rep. 2025 Apr 22;15:13992. doi: 10.1038/s41598-025-96569-4 (PMC12015547; doi:10.1038/s41598-025-96569-4)
Supplement: Supplementary file 1 — Supplementary Information. [file 41598_2025_96569_MOESM1_ESM.pdf]

Supplementary Table S1. Missingness Proportion per Variable (%).

Variable	Agincourt	Nairobi
age	0	0
alcohol use status	0	0.11
BMI	0.8	0
cholesterol	0	0
CIMT mean max	12.71	0.17
friedewald LDL	0.15	0.51
HDL	0	0
highest level of education	0	0
hiv final status	0	0
MVPA (categorical)	0	0
occupation	5.59	0.17
partnership status	0	0.06
SES site quintile	0	0
sex	0	0
smoking status	0.15	0.06
subcutaneous fat	12.27	0.11
triglycerides	0	0
visceral fat	15.69	0.11
waist circumference	0.51	0.28
waist hip ratio	0.58	0.28

Supplementary Algorithm S1: Pseudo-code for Multi-dimensional subset scanning (MDScan)

```
1  # Input and output definition;
   input  : Dataset: 𝒟 = {(x_i, y_i) | i = 1, 2, ⋯ , N}, Set of features: ℱ = [f_1, f_2, ⋯ , f_m, ⋯ , f_M]
   output : AnomSubset,
            AnomScore
2  # Initialization ;
3  AnomSubset ← {};
4  AnomScore ← −∞;
5  UnCheckedF ← ℱ;
6  #Iterate until convergence;
7  while UnCheckedF isNot {} do
8      # Randomly select unchecked feature;
9      f_m ← Random(UnCheckedF);
10     # Mark the feature as checked;
11     UnCheckedF ← UnCheckedF \ f_m;
12     # Compute the anomalous score;
13     Score, Subset ← ALTSS (f_m | AnomSubset);
14     # Compare the new score with previous best;
15     if Score > AnomScore then
16         # Update the score, subset and reset the flag to unchecked;
17         AnomScore ← Score ;
18         AnomSubset ← Subset ;
19         UnCheckedF ← ℱ;
20     else
21         Go to Step 4 ;
22     end
23 end
24 # Return the most anomalous score and its subset;
25 return AnomSubset, AnomScore
```

Supplementary Table S2. Subpopulations of participants in Agincourt (n=1377; 16.4% multimorbidity) with high and low risk for multimorbidity as identified by Automatic Stratification for smaller feature space. We use the same feature space as in the main experiments but exclude the following features - visceral fat, subcutaneous fat, CIMT mean max, HIV status, and partnership status. The first four were excluded because they may not be easy to get in a public health setting while the last one was excluded because it could change rapidly.

Risk Status	NL	Subpopulation Description	OR	P(S)	P(MM|S)	P(S|MM)
High risk	2^{++}	age $\geq$ 54 & waist circumference $\geq$ 980	3.67	0.114	0.369	0.257
	3^{++}	age $\geq$ 54 & BMI $\geq$ 23.43 & waist hip ratio $\geq$ 0.86	3.48	0.177	0.336	0.363
	4^{++}	age $\geq$ 54& BMI $\geq$ 23.43 & waist circumference $\geq$ 830 & waist hip ratio $\geq$ 0.86	3.58	0.174	0.342	0.363
Low Risk	2^{++}	cholesterol $\leq$ 4.41 & waist circumference $\leq$ 830	0.31	0.252	0.069	0.106
	3^{++}	Friedewald LDL $\leq$ 2.753 & occupation in [4]) & waist hip ratio $\leq$ 0.93	0.28	0.293	0.067	0.119
	4^{++}	HDL $\leq$ 1.28 & MVPA in [1.0] & occupation in [4] & waist circumference $\leq$ 830	0.07	0.096	0.015	0.009

NL refers to the number of literals present in the description, OR refers to the odds ratio of the described subpopulation. P(S) refers to the size of the subpopulation as a proportion of the overall size of the dataset. P(MM|S) refers to the proportion of the described subpopulation that have multimorbidity. P(S|MM) refers to the proportion of multi-morbid people in the entire population that the described subpopulation covers. $\{NL\}^{++}$ means subset was statistically significant at $p-value = 0.01$.

Supplementary Table S3. Subpopulations of participants in Nairobi (n=1777; 9.7% multimorbidity) with high and low risk for multimorbidity as identified by Automatic Stratification for smaller feature space. We use the same feature space as in the main experiments but exclude the following features - visceral fat, subcutaneous fat, CIMT mean max, HIV status, and partnership status. The first four were excluded because they may not be easy to get in a public health setting while the last one was excluded because it could change rapidly.

Risk Status	NL	Subpopulation Description	OR	P(S)	P(MM|S)	P(S|MM)
High risk	2^{++}	age $\geq$ 51 &<br>waist circumference $\geq$ 920	3.73	0.12	0.238	0.297
Low Risk	2^{++}	age $\leq$ 45 &<br>waist hip ratio $\leq$ 0.91	0.24	0.222	0.3	0.07

NL refers to the number of literals present in the description, OR refers to the odds ratio of the described subpopulation. P(S) refers to the size of the subpopulation as a proportion of the overall size of the dataset. P(MM|S) refers to the proportion of the described subpopulation that have multimorbidity. P(S|MM) refers to the proportion of multi-morbid people in the entire population that the described subpopulation covers. $\{NL\}^{++}$ means subset was statistically significant at $p-value=0.01$

Supplementary Table S4. We rerun the main experiments with a further sex stratification after the site stratification to understand the sex differences in multimorbidity patterns. Subpopulations of female participants in Agincourt (n=832; 18.1% multimorbidity) with high and low risk for multimorbidity as identified by Automatic Stratification.

Risk Status	NL	Subpopulation Description	OR	P(S)	P(MM|S)	P(S|MM)
High risk	2^{+}	age $\geq$ 48 & waist hip ratio$\geq$ 0.93	2.834	0.133	0.317	0.257
	4^{+}	age $\geq$ 54 & Friedewald LDL $\geq$ 2.881 & waist circumference $\geq$ 870 & waist hip ratio $\geq$ 0.86	4.831	0.051	0.457	0.142
Protective	2^{+}	Friedewald LDL $\leq$ 2.881 & waist hip ratio $\leq$ 0.93	0.518	0.286	0.107	0.186
	3^{-}	cholesterol $\leq$ 4.58 & occupation in [4] & waist hip ratio $\leq$ 0.93	0.35	0.179	0.073	0.08

NL refers to the number of literals present in the description, OR refers to the odds ratio of the described subpopulation. P(S) refers to the size of the subpopulation as a proportion of the overall size of the dataset. P(MM|S) refers to the proportion of the described subpopulation that have multimorbidity. P(S|MM) refers to the proportion of multi-morbid people in the entire population that the described subpopulation covers. $\{NL\}^{+}$ means subset was statistically significant at $p-value=0.05$ while $\{NL\}^{-}$ means subset was statistically insignificant at $p-value=0.05$.

Supplementary Table S5. We rerun the main experiments with a further sex stratification after the site stratification to understand the sex differences in multimorbidity patterns. Subpopulations of male participants in Agincourt (n=545; 13.8% multimorbidity) with high and low risk for multimorbidity as identified by Automatic Stratification.

Risk Status	NL	Subpopulation Description	OR	P(S)	P(MM|S)	P(S|MM)
High risk	3^{++}	subcutaneous fat $\geq$ 1.47 & age $\geq$ 54 & highest level of education in [1]	9.021	0.017	0.625	0.066
	4^{++}	triglycerides $\geq$ 0.63 & subcutaneous fat $\geq$ 1.47 & age $\geq$ 54 & highest level of education in [1]	17.501	0.012	0.765	0.058
Protective	2^{++}	Friedewald LDL $\leq$ 2.398 & 0.749 $\leq$ waist hip ratio $\leq$ 0.87	0.098	0.103	0.021	0.013
	4^{++}	CIMT mean max $\leq$ 0.72 & MVPA in [1.0] & SES site quintile in [3 , 4]	0.041	0.083	0.009	0.004

NL refers to the number of literals present in the description, OR refers to the odds ratio of the described subpopulation. P(S) refers to the size of the subpopulation as a proportion of the overall size of the dataset. P(MM|S) refers to the proportion of the described subpopulation that have multimorbidity. P(S|MM) refers to the proportion of multi-morbid people in the entire population that the described subpopulation covers. $\{NL\}^{++}$ means subset was statistically significant at $p-value=0.01$.

Supplementary Table S6. We rerun the main experiments with a further sex stratification after the site stratification to understand the sex differences in multimorbidity patterns. Subpopulations of female participants in Nairobi (n=962; 11.4% multimorbidity) with high and low risk for multimorbidity as identified by Automatic Stratification.

Risk Status	NL	Subpopulation Description	OR	P(S)	P(MM|S)	P(S|MM)
High risk	2^{++}	waist hip ratio $\geq$ 0.91 & age $\geq$ 50	4.763	0.077	0.294	0.233
	4^{++}	waist hip ratio $\geq$ 0.85 & triglycerides $\geq$ 1.13 & visceral fat $\geq$ 3.96 & age $\geq$ 50	7.481	0.05	0.398	0.203
Protective	3^{++}	waist hip ratio $\leq$ 0.91 & age $\leq$ 50 & waist circumference $\leq$ 837.0	0.112	0.125	0.014	0.017

NL refers to the number of literals present in the description, OR refers to the odds ratio of the described subpopulation. P(S) refers to the size of the subpopulation as a proportion of the overall size of the dataset. P(MM|S) refers to the proportion of the described subpopulation that have multimorbidity. P(S|MM) refers to the proportion of multi-morbid people in the entire population that the described subpopulation covers. $\{NL\}^{++}$ means subset was statistically significant at $p-value = 0.01$.

Supplementary Table S7. We rerun the main experiments with a further sex stratification after the site stratification to understand the sex differences in multimorbidity patterns. Subpopulations of male participants in Nairobi (n=815; 7.6% multimorbidity) with high and low risk for multimorbidity as identified by Automatic Stratification.

Risk Status	NL	Subpopulation Description	OR	P(S)	P(MM|S)	P(S|MM)
High Risk	4^{--}	subcutaneous fat $\leq$ 1.28 & CIMT mean max $\geq$ 0.72 & highest level of education in [1]) & triglycerides $\geq$ 1.16	8.523	0.015	0.462	0.07
Low Risk	2^{-}	HDL $\leq$ 1.33 & triglycerides $\leq$ 0.75	0.155	0.095	0.018	0.017
	3^{-}	HDL $\leq$ 1.33 & BMI $\leq$ 23.97 & triglycerides $\leq$ 0.75	0	0.068	0	0

NL refers to the number of literals present in the description, OR refers to the odds ratio of the described subpopulation. P(S) refers to the size of the subpopulation as a proportion of the overall size of the dataset. P(MM|S) refers to the proportion of the described subpopulation that have multimorbidity. P(S|MM) refers to the proportion of multi-morbid people in the entire population that the described subpopulation covers. $\{NL\}^{-}$ means subset was statistically insignificant at $p-value = 0.05$ while $\{NL\}^{--}$ means subset was statistically insignificant at $p-value = 0.1$.
